# Current Challenges in Vaccinology

**DOI:** 10.3389/fimmu.2020.01181

**Published:** 2020-06-25

**Authors:** Richard B. Kennedy, Inna G. Ovsyannikova, Peter Palese, Gregory A. Poland

**Affiliations:** ^1^Mayo Clinic Vaccine Research Group, Mayo Clinic, Rochester, MN, United States; ^2^Icahn School of Medicine at Mount Sinai, New York, NY, United States

**Keywords:** vaccine, genomics, transcriptomics, vaccine development, genetics

## Abstract

The development of vaccines, which prime the immune system to respond to future infections, has led to global declines in morbidity and mortality from dreadful infectious communicable diseases. However, many pathogens of public health importance are highly complex and/or rapidly evolving, posing unique challenges to vaccine development. Several of these challenges include an incomplete understanding of how immunity develops, host and pathogen genetic variability, and an increased societal skepticism regarding vaccine safety. In particular, new high-dimensional omics technologies, aided by bioinformatics, are driving new vaccine development (vaccinomics). Informed by recent insights into pathogen biology, host genetic diversity, and immunology, the increasing use of genomic approaches is leading to new models and understanding of host immune system responses that may provide solutions in the rapid development of novel vaccine candidates.

## Introduction

Infectious diseases can lead to illness, human suffering, economic costs, medical complications, hospitalization, disability, and death. Besides sanitation and clean water, vaccines have had the greatest impact on human health and longevity (1). The cost of vaccine-preventable diseases (VPD) just in the USA during 2015 was estimated at $9 billion (2). From 2011–2020, one model estimated that 23.3 million deaths worldwide will have been averted by vaccines (3). In the 2017–2018 influenza season, it is estimated that almost one million Americans were hospitalized and 90,000 died due to influenza (4). Worldwide, it is estimated that, between 2000 and 2014, 17.1 million deaths due to measles were averted by the use of the measles vaccine (5).

While there are over 1,400 known species of human pathogens with more being discovered every year, in the US, licensed vaccines exist for only 26 pathogens (6). Preventing infections with vaccines is a complex, costly, and lengthy process that requires overcoming multiple challenges before resulting in a safe and effective product (Box 1). Historically, vaccine development has followed an empiric “isolate, inactivate and inject” paradigm, whereby the disease-causing pathogen or its disease-mediating entity (e.g., a toxin) is identified, inactivated, and injected in order to elicit a protective immune response (7–9). This empiric method, developed before the genetic revolution, enabled the development of many early and effective vaccines against pathogens such as influenza, tetanus, diphtheria, and pertussis. In the case of viruses, including smallpox, measles, mumps, rubella, and smallpox, a parallel approach has been to substitute inactivation with attenuation.

Box 1 **Current Challenges Facing Vaccine Development Efforts.**

The creation of new vaccines is a slow, systematic, expensive, and laborious process that requires coordination between scientists, physicians, public health officials, industry and vaccine developers, and society. These shareholders must work together in order for us to overcome the listed challenges in order to successfully development safe and effective vaccines that see widespread use.

- High (and increasing) costs for vaccine development (~$700 million–$1 billion)
- Vaccine hesitancy
- More stringent safety requirements
- Societal expectations of 100% efficacy
- Need to maintain cold-chain for vaccines
- Increasing requirements for single dose efficacy
- Need for rapid response to global outbreaks
- Limited number of vaccine manufacturers
- Product development time (typically ~10 years)
- Current pathogens require more complicated vaccines
- Low efficacy of some licensed vaccines
- Business models prioritize vaccines by market potential, not by public health need
- Aging world population that respond poorly to most vaccines (immunosenescence)
- Limited number of approved and acceptable adjuvants
- Concurrent health problems in developing world that compromise immune response (nutrition, co-infection)
- Incomplete or inadequate understanding of biology, pathogenesis, and/or immunology of emerging pathogens
- Inability to properly attenuate pathogens OR risk of reversion to wild type organism
- Humoral immune responses do not always correlate with protection
- Inappropriate/harmful immune response (formalin-inactivated RSV products) or enhanced disease upon re-infection (Dengue)
- Inadequate durability of immune response (ex. Pertussis)

**Figure 1:**
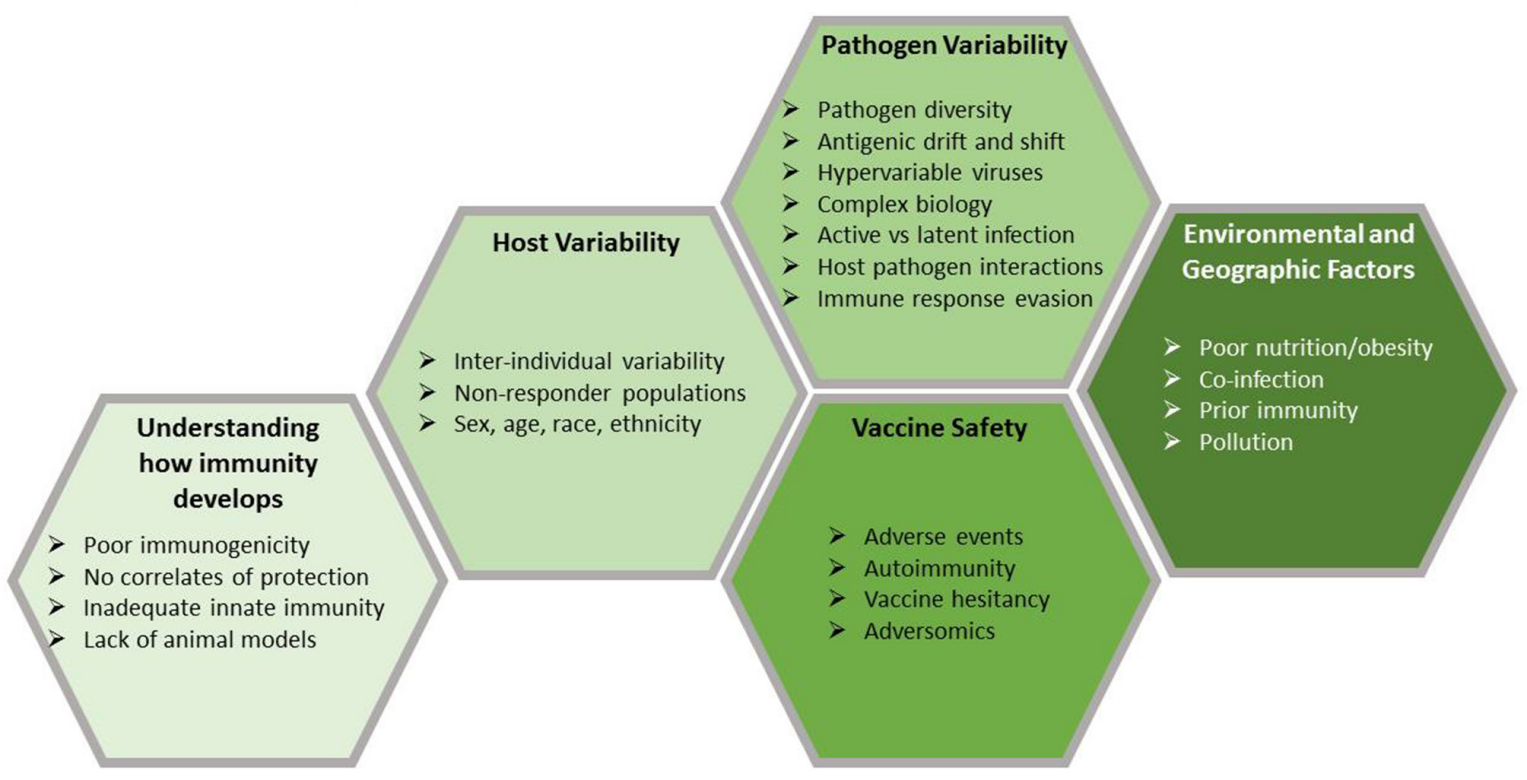
Barriers to vaccine development. Vaccines are the most effective public health tool for controlling infectious diseases. Despite considerable success, there is room for improvement in many current vaccines and there are a large number of new and re-emerging pathogens for which we do not have effective vaccines. Vaccine development faces a number of challenges, many of which are presented here. Developing vaccines to combat current and future pathogens will require us to overcome those challenges and recent developments in genomic technologies may provide the solutions that we need.

While this empiric approach has led to tremendous successes, the work is far from finished; major, significant barriers remain (Figure 1). This review focuses on five of these barriers: an incomplete understanding of how immunity develops, host and pathogen genetic variability, problems related to vaccine safety, and both environmental (e.g., nutrition, obesity) and geographic factors (e.g., maintaining a cold chain in Sub-Saharan Africa, co-infection in tropical climates) that compromise vaccine usage or efficacy. Because of these barriers, the traditional empiric approach has been ineffective for developing vaccines against hypervariable and highly complex pathogens, such as *Mycobacterium tuberculosis*, malaria-causing *Plasmodium*, hookworm, HIV, HCV, coronaviruses, among others. This is due to their complex life cycles, the ability of these pathogens to rapidly alter their surface proteins (i.e., antigenic variation) and other mechanisms by which the pathogen can evade host detection and the host immune response. Complex immunology can also be a barrier; for example, the recent demonstration of antibody-dependent disease enhancement which has hindered the development and use of the recently licensed Dengue vaccine (10).

**Figure 2:**
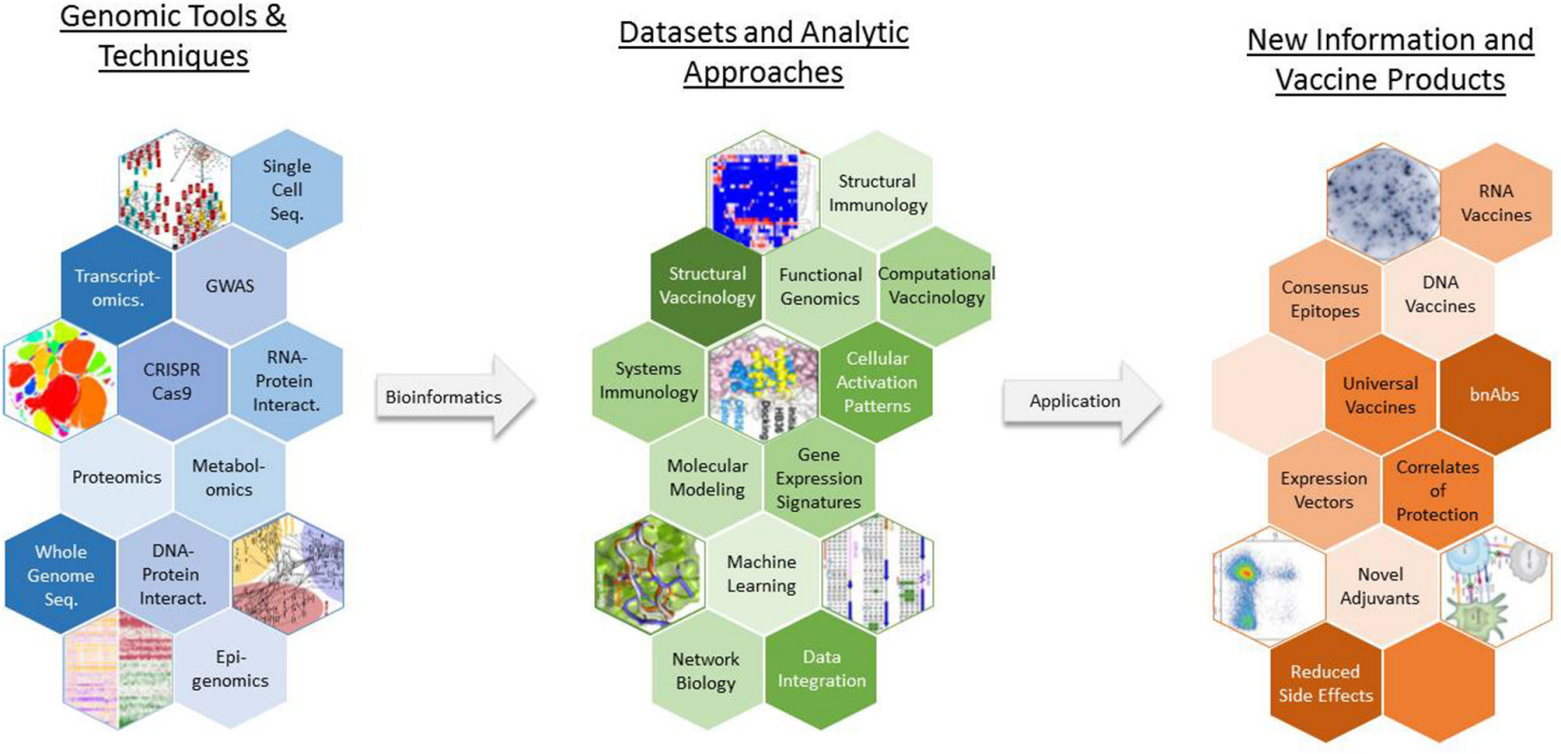
The use of vaccinomics in new vaccine development. A wide array of genomic tools and techniques are available for researchers to study various aspects of pathogen biology and host physiology and immune response. Vaccinomics and similar approaches represent toolboxes that contain a specific assortment of laboratory assays, statistical analysis routines, bioinformatic methods, and computational models that can be used to generate an appropriate dataset and extract biologically meaningful results from the data. GWAS, genome wide association study; bnAbs, broadly neutralizing antibodies.

In response to the challenges posed by these barriers, novel approaches such as vaccinomics (which aims to understand genomic and systems-level data to elucidate the basis of inter-individual variations in immune responses), reverse vaccinology (which uses genetic sequence information to identify immunogenic antigens), and structure-based vaccine design have been developed to take advantage of high-dimensional tools and techniques and generate novel data that can be leveraged to create new vaccine products (Figure 2) (11–13). In the past decade, new vaccines, including the licensed Meningococcus B vaccine, have been designed and developed using such genomics-based approaches (14, 15). With the increasing sophistication and decreased expense of gene-based assays and next-generation sequencing technologies, genomics is accelerating the development of new vaccines in the twenty-first century—closely paralleling the application of genomics to other aspects of human medicine, such as individualized medicine.

Genetics has expanded far beyond the simple nucleic acid sequence of a given organism. While it primarily deals with individual genes, it also includes the myriad regulatory mechanisms that control gene expression (16–22). Similarly, genomics has also expanded in scope to include the comprehensive characterization of gene expression, regulation, interdependency, pre- and post-transcriptional modifications, gene editing, epistasis, complementarity, pleiotropy, and other complex interactions (23).

**Figure 3:**
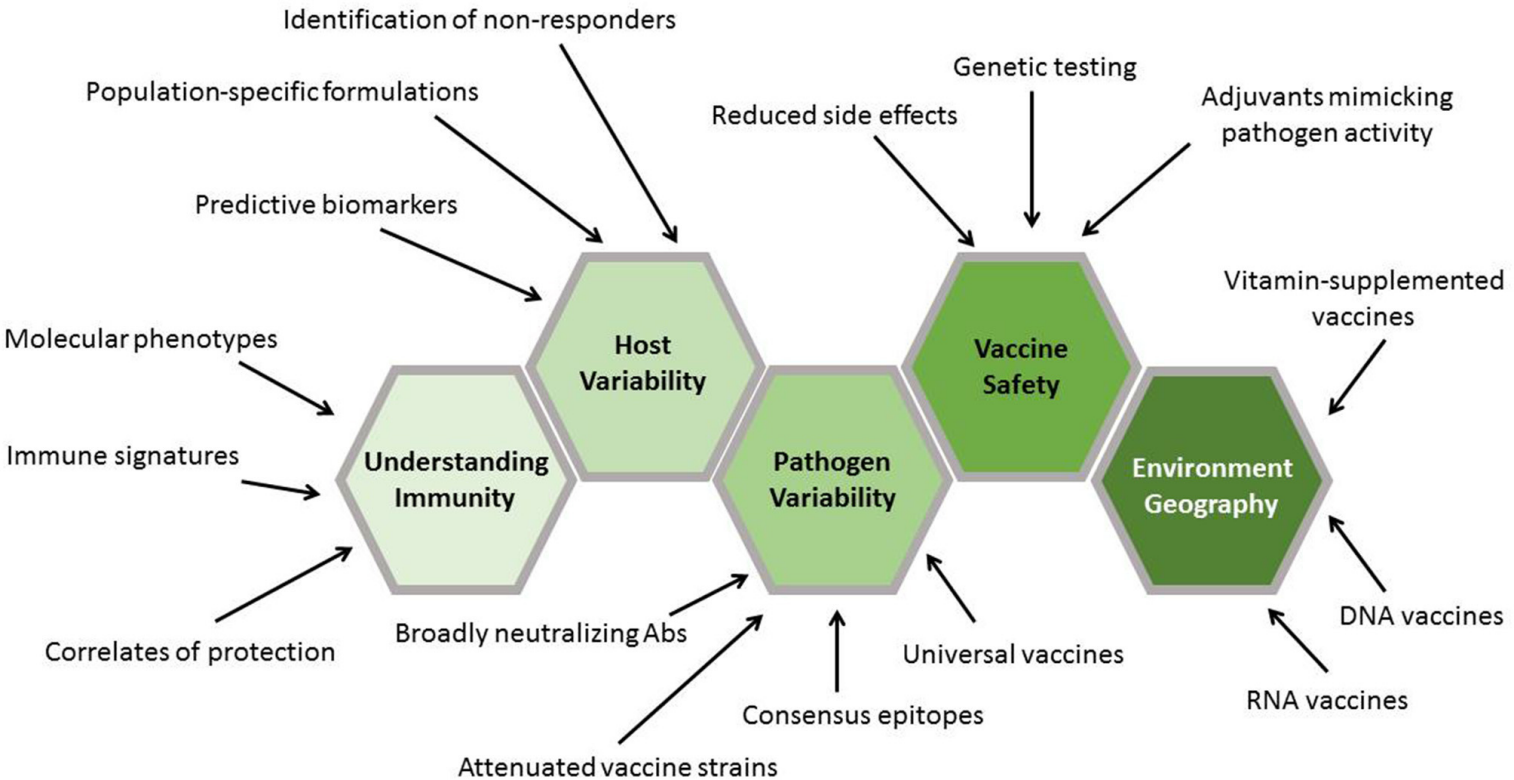
Contribution of genomics to vaccine development stages. The results generated by genomic approaches inform each stage of vaccine development. Initial genomics work plays a critical role in discovering new vaccine targets. This is followed by the characterization of these targets in terms of their ability to generate protective immune responses. Validation of the findings in genetically diverse populations is aided by information gained through genomics approaches. Finally, application of the findings assists in the design and completion of phase I–IV clinical trials.

Genomics is not the only area that has undergone remarkable transformation recently in terms of the technologies and platforms that can be used to design, create, and study vaccines. Examples include the following: mass cytometry, which allows for incredibly complex immunophenotyping (24, 25); proteomics and mass spectrometry (26–30); and metabolomics, which has been closely linked to immunologic function and vaccine response (31–33). However, in this focused review, we will explore how genomics and recent genomic technologies have impacted vaccine development and may provide solutions to both the long-standing barriers in vaccine design and the new challenges posed by new and re-emerging pathogens of public health importance. Creative application of these tools and the biological insights that they provide are poised to truly revolutionize how we design, develop, test, and deploy vaccines (Figure 3).

## Barrier #1: Incomplete Understanding of How Immunity Develops

The human immune system is incredibly complex, with multiple tissues and organs, dozens of different signaling pathways (34), hundreds of different cells (35), thousands of different effector molecules, and an effectively infinite ability to recognize foreign antigens (36)—all of which must be “choreographed” effectively, kinetically, and in proper sequence. Immunologists have developed a large and comprehensive (but by no means complete) catalog of the individual parts that make up the system (37–39); however, our reductionist understanding of how these parts collaboratively function as a “system” has lagged behind (40). While we understand what most of the parts do individually, we have more trouble understanding how each component inter-relates and collectively contributes to the development of immunity at the systems-level (41, 42). In short, we do not comprehensively understand the rules governing the behavior of the system and therefore cannot reliably and consistently predict the outcome of a given infection or vaccination (43). Developing this understanding is a critical first step in our ability to predict and eventually manipulate the immune system in order to achieve the desired outcome of protective immunity (44).

This is the knowledge gap that systems biology and vaccinomics paradigms seek to fill by capturing complex relationships among immune components as the host responds to infection or vaccination—rather than simple reductionist approaches to single components of the system. These studies have been made possible by our growing ability to measure increasingly larger and more complete collections of molecules. For example, instead of a single quantitative PCR reaction, we can use next generation sequencing to simultaneously sequence millions of different DNA or RNA molecules (45, 46). We can characterize comprehensive changes within each cell's entire transcriptome, epigenome, proteome, metabolome, and multiple other “-omes” (47–49). We can characterize the cell types and sub-types involved in the response, their phenotype, their activation state, and their biological functions (50, 51). We can capture the signals generated by multiple signal-transduction pathways inside each cell (52) and observe the communication occurring between cells in normal health (53) and during infection (54). Collectively, these technologies have been applied to understanding immune function, host-pathogen interactions, pathogen genetics, and pathogenesis in unprecedented detail (55–58).

A central organizing feature of these efforts is a focus on genomics, as gene expression is considered a critical first step because immune cells recognize and react to foreign antigens. Consequently, each of these studies has a primary goal of understanding the transcriptomic changes that occur during the development of an immune response. Querec et al. first applied high-throughput data to the characterization of yellow fever vaccine response in humans (59). Systems analysis was used to discover a distinct molecular signature that predicted the neutralizing antibody (Ab) (i.e., *TNFRS17* gene signature) and antigen-specific CD8+ T cell (i.e., *C1QB* and *EIF2AK3* gene signature) responses to the live attenuated yellow fever vaccine YF-17D in humans with up to 100 and 90% accuracy, respectively (59). Because expression levels of the genes identified in this study were highly predictive of both humoral and cell-mediated immune responses, these signatures can potentially function as early biomarkers of vaccine response, efficacy, and even safety. Similarly, Dunachie et al. identified a gene expression signature that correlates with vaccine-induced protection in a human malaria challenge model in which the expression of genes associated with IFN induction and with antigen presentation correlated with protection against malaria (60).

Transcriptomic studies can reveal important factors controlling disease susceptibility and clinical outcomes during infection or vaccination. Through mechanisms that are not fully understood, clinical symptoms of dengue virus infection range from asymptomatic or mild disease (80%) to severe, life-threatening dengue hemorrhagic fever (DHF) or dengue shock syndrome (DSS). Transcriptomic profiling of the central nervous system (CNS) of mice infected with dengue identified putative innate signaling pathways (IFN signaling, IL-10, GM-CSF, PDGF), antigen processing, and complement activation signatures, which suggests that innate immune responses may serve to limit dengue virus replication in the CNS and thereby reduce disease severity (61). These findings suggest that adjuvant-mediated activation of these pathways could enhance vaccine response and/or provide therapeutic benefit. Similar gene expression studies in humans with dengue illness suggest that a transcriptomic signature detectable as early as 1 day after infection can potentially distinguish between dengue fever and the more serious dengue hemorrhagic fever (62). These results inform the development of molecular diagnostics and treatment options for patients.

Ebola virus infection is another disease where pathogenesis is not completely understood and transcriptomic analysis has revealed important insights into Ebola disease progression. Non-human primate survivors of experimental infection displayed upregulation of specific genes, including CCL8, compared to animals that succumbed to infection (63). Although the study was focused on therapeutics, the findings suggest additional correlates of protection beyond the typical antibody measures. In yet another example, microarrays have also been used to identify gene expression patterns (i.e., upregulation of NF-kB and IFNg signaling) that correlate with protection in trials with the malaria RTS,S vaccine (64). Thus, studies evaluating transcriptomic changes after infection/vaccination have provided rich insights into mechanisms of disease initiation, clinical progression, and vaccine-induced immunity (65). These studies have also identified potential correlates of protection and yielded predictive biomarkers that can be used to inform clinical care or to provide early go/no-go criteria for vaccine trials.

Systems biology studies have also provided important insights into the generation and maintenance (i.e., durability) of immune responses to many vaccines, including seasonal influenza (trivalent inactivated influenza vaccine [TIV] and MF59-adjuvanted influenza vaccine), malaria (RTS,S), meningococcal (MPSV4 and MCV4), and others (66–70). A systems biology approach comparing MF59-adjuvanted and TIV vaccine in immune-immature children (14–24-months-old, *n* = 90) identified significantly higher transcriptional responses to the MF59-adjuvanted vaccine and identified early innate response signatures correlated with Day 28 Ab titers (67). These include M16 (a module associated with TLR and inflammatory signaling); M11 (a module regulating monocyte function); M75 (a module controlling IFN-induced antiviral response); M156 (a module associated with Ab secreting cells); and S3 (a module with genes involved in immunoglobulin production). These findings may provide potentially generalizable molecular correlates of Ab production during early childhood (67).

Several adjuvants, such as MF59, AS01-4, TLR9 agonists, virosomes, and others have recently been licensed for use in human vaccines. For example, a recent Hepatitis B vaccine (Heplisav B) incorporating a TLR9 agonist has considerably improved seroconversion rates compared to other hepatitis vaccines—particularly in subjects who normally respond poorly and slowly (71). Similarly, the recently FDA-licensed MF59-adjuvanted influenza subunit vaccine (72) induces higher antibody titers, a broader humoral response, and longer persistence of influenza Ab titers than the non-adjuvanted, standard-dose influenza vaccine in older adults. This is a population that suffers the greatest burden of influenza-associated morbidity and mortality, yet has the poorest response to standard influenza vaccines (73, 74). A recent large study in 7,082 individuals (≥65 years of age) demonstrated significantly higher immunogenicity (*p* < 0.001, seroconversion and HAI GMT) of the MF59-adjuvanted vaccine compared to standard dose influenza vaccine (75). Similarly, the diversity, commonalities, and differences in human genetic and immune responses to two varicella zoster virus (VZV) vaccines, the live attenuated vaccine (Zostavax), and the AS01B-adjuvanted glycoprotein E vaccine (Shingrix) are being examined using systems biology approaches (31, 76). These vaccines exhibit significant differential immunogenicity and significant variations in the longevity of immunologic memory. Furthermore, the effect of age and immunosenescence is drastically different with these two vaccines. These clinically important differences provide an ideal system for studying the systems-level factors contributing to these differences and are likely to drastically improve our understanding of zoster immunology. In fact, recent reports have highlighted the novel finding that the magnitude and durability of immune responses to zoster vaccination are dependent on the abundance of both regulatory T cells and T cells expressing checkpoint markers (e.g., PD-1) (77). Systems studies examining the durability of immunity after measles, mumps, and rubella vaccination are in progress (78–84).

### Structural Vaccinology

Structural biology studies have allowed investigators to map viral epitopes onto the three-dimensional structure of pathogen proteins (85, 86). Antibody-antigen complexes can also be determined, providing insights into critical antibody functionality such as neutralization or have identified critical conserved regions that can be targeted for more effective immune responses (87). Insights into conformational changes with RSV have enabled investigators to develop new vaccines that avoid the limitations of historical approaches (88) and design better immunogens (89). Another excellent example of structural vaccinology is the increasing use of virus-like-particles as vaccine platforms (90, 91).

## Barrier #2: Host Variability

### Genome-Wide Association Studies (GWAS)

Inter-individual variation in vaccine-specific immune response is known to be influenced by host gene polymorphisms. This genetic variability of the human population gave rise to vaccine-immunogenetics research focused on finding important genetic variants associated with variations in immune responses by assessing relationships between variability in immune response to vaccines and genetic factors. Certainly, population-based candidate gene association studies of vaccine-specific immune responses are beginning to reveal and explain how—and to what degree—variations in innate and adaptive immune responses following vaccination are determined by gene polymorphisms (92, 93). While a candidate gene approach was thought most efficient in the past decade, it is clear that a GWAS is an unbiased, agnostic approach that serves as a critical step in the research by identifying genetic variants impacting immunity and supporting a novel paradigm by which vaccine development could occur (92, 94, 95). A GWAS allows the identification of individual and groups of genes and genetic variants (SNPs, or single nucleotide polymorphisms) that are associated with specific markers of vaccine-induced immunity. At the systems level, genotype/phenotype computational models that integrate numerous additive and epistatic marker effects are needed. The evidence thus far suggests that the effect of one gene/allele depends on the presence of another gene/allele that may control a phenotype (e.g., epistatic interactions). The integration of epistasis network analysis and functional interactions into genotype-phenotype association studies have provided important insights into smallpox vaccine-induced immunity and specifically the role of variants in *RXRA* (the gene encoding a vitamin A receptor) in immune responses to smallpox and other viral vaccines (96–98). The most thorough and efficient study for such purposes is a two-stage (discovery–replication) genome-scale analysis (99), followed by functional studies to (1) validate which specific gene polymorphisms and pathways/gene sets have the biggest or most critical effect on inter-individual variations in immune responses among immunized subjects, and (2) identify the mechanism(s) by which these effects occur. Significant work delineating the effect of gene polymorphisms on hepatitis B, measles, mumps, rubella, influenza, smallpox, and anthrax vaccine-induced immune responses has been published (98, 100–107). Examples include the identification and replication of a CD46 measles virus receptor variant coding for a 53% reduction in Ab response to measles vaccine, which is a finding that could be used to reverse engineer a vaccine to circumvent this viral receptor genetic restriction (108). Our studies identified a SNP (rs2064479, *p* = 8.6 × 10^−8^) in the class II HLA-DPB1 gene region associated with variations in rubella-specific Ab titers after rubella vaccine (106). Additional SNPs (*p* ≤ 1.0 × 10^−7^) in high linkage disequilibrium (LD, r^2^ ≥ 0.8) of rs2064479 were also positioned near the genetic region of HLA-DPB1. Some of these polymorphisms were predicted to be located in miRNA binding sites. These data validate the previous findings of HLA-DPB1 genotypes (i.e., HLA-DPB1^*^04:01 and HLA-DPB1^*^03:01) linked with rubella vaccine-specific immune response (109, 110). It has been previously demonstrated that the DPB1^*^04:01 and DPB1^*^03:01 alleles are associated with significantly higher and lower Ab responses, respectively (109, 110). It is highly likely that the DPB1^*^04:01 molecule presents an array of processed epitopes to CD4+ T cells different from that of the DPB1^*^03:01 allele and is therefore able to stimulate more robust rubella-specific T cell responses, which in turn elicit robust humoral immune responses. Indeed, earlier work revealed that HLA genes/proteins are critical elements for immune responses to rubella vaccination, accounting for ~20% of the total genetic inter-individual variation in Ab response to rubella (109).

Multiple GWAS studies have shown that allele-specific HLA class I and class II genetic polymorphisms play a fundamental function in the differential generation of viral vaccine-induced immune responses (109, 111–116). Identifying which specific HLA alleles are associated with protective immune responses through vaccination is critical for population health and for a deeper understanding of vaccine-induced immunology and vaccine development. We have leveraged such knowledge to identify naturally processed and HLA-presented viral-derived epitopes using mass spectrometry techniques (117). Peptide identification using this approach provides the framework for the selection and use of these immunodominant pathogen epitopes as candidate vaccine targets (118). Studies have illustrated several regulatory and common SNPs in the different regions of HLA genes associated with immune responses to childhood immunization, such as the capsular group C meningococcal (MenC); Haemophilus influenza type B (Hib); tetanus toxoid (TT); hepatitis B (HBV); 7-valent pneumococcal conjugate (PCV7); and the diphtheria, tetanus, and acellular pertussis (DTaP) vaccines (119, 120). Through studies such as these, GWAS can be used to identify critical genetic determinants of vaccine-specific immunity and assist in the development of novel vaccines that overcome these genetic restrictions.

GWAS studies also demonstrate that multigenic effects (121, 122) including HLA and a variety of immune, innate, and adaptive gene SNPs significantly affect immune responses to vaccines (114, 123–126). Likewise, synergistic effects of tapasin gene polymorphisms and specific HLA class I alleles to generate stronger anti-viral CD8+ T cell responses have been observed. In a study of subjects with resolved or chronic hepatitis C virus (HCV) infection in UK, Germany and US, tapasin G alleles in a combination with specific HLA class IB alleles with an aspartate (Asp) at residues 114 and 156 have been associated with stronger anti-viral CD8+ T cell responses against HCV and with the outcome of HCV infection (127). This suggests that tapasin gene polymorphisms maybe important for antigen processing and HLA class I peptide loading mechanisms (128). A large number of other genes and gene families (e.g., interferon response factors, pattern recognition receptors, cytokines, chemokines) have been implicated in the control of immune responses to vaccines (129), and the literature is full of disease susceptibility studies that highlight additional genes and pathways contributing to immune responses to pathogens (130). Many of these study results are available in online databases such as this one: https://www.ebi.ac.uk/gwas/. Genetic studies of vaccine responses have revealed effects that are both quantifiable and predictable (7, 13, 109, 114, 121, 122, 131–135). Informed by such studies, the development of novel vaccines and adjuvants that specifically target innate receptors and their signaling pathways (e.g., TLR pathway), leading to higher protection rates and enhanced immune responses, is possible.

## Barrier #3: Pathogen Variability

Pathogen genetic sequence variability is a major impediment to vaccine development (136–138). This can manifest in multiple ways: (1) tremendous sequence diversity among viral strains—as an example, a major challenge in the development of an effective rhinovirus vaccine is that it must elicit cross-protective immunity across over 160 different circulating rhinovirus strains (139); (2) antigenic drift or shift, as demonstrated by influenza viruses, which necessitates a yearly reformulation of influenza vaccines; (3) a complicated lifecycle during which large segments of the genome are turned on and off, as is the case with *Plasmodium falciparum* (Plasmodium life-cycle stages also affect the type of immune response that is required to combat the pathogen); (4) pathogens with large, complex genomes, such as Mycobacterium tuberculosis, where effective immunologic targets or immunomodulatory molecules are difficult to identify, and therefore have not been effectively dealt with; (5) vaccine-induced pressure leading to changes in serotype prevalence, as has been demonstrated with the heptavalent pneumococcal vaccine (140); (6) pathogens with rapid mutation rates, such as HIV and HCV, also complicate the issue as the antigenic targets of the immune response rapidly shift during an infection forcing the immune response to chase an ever-changing target; (7) zoonoses that cross the species barrier to infect humans (e.g., SARS-CoV, MERS-CoV, H5, SARS-CoV-2).

The host immune response typically recognizes and responds to a small set of immunodominant epitopes (141). For humoral responses, these epitopes are typically the linear or conformational areas that are readily accessible to antibodies. Unfortunately, these areas of the pathogen genome are often hot spots for mutation or recombination events, enabling the pathogen to evade immune responses by displaying modified surface proteins that are no longer recognized by existing antibodies, forcing the immune system to start over—an effect repeatedly demonstrated by influenza virus and HIV (142–145). Sequence differences between viral, bacterial, and parasite strains are often found at these locations; therefore, a neutralizing antibody specific to an epitope on the HA protein of one influenza strain will not necessarily bind to or neutralize that same site on another influenza strain. An analogous situation exists for bacteria, where a second strain may possess entirely different virulence factors than the first. The new strain may be effectively invisible to the immune response specific for the first strain. In this manner, strain diversity contributes to antigenic differences that determine whether or not immune responses are cross-protective. Understanding the factors controlling immunodominance and how pathogens exploit this is of critical importance (146, 147).

By identifying genetically conserved regions, investigators can target epitopes more likely to be present across multiple strains, thereby creating immune responses that are cross-protective. For example, the use of conserved stalk regions of the influenza hemagglutinin (HA) protein to develop universal influenza vaccines is an excellent example of this type of work. Another example is the *Plasmodium falciparum* Reticulocyte Binding Protein Homolog 5 (PfRh5), which facilitates parasite entry into human red blood cells through binding to the Ok blood group antigen (148). Because the PfRh5 protein is targeted by broadly acting, parasite-neutralizing antibodies that transcend different strains, PfRh5-based vaccines have shown promise as vaccine immunogens (149).

Genome-sequence data is used to do the following: determine pathogen strain diversity; identify virulence factors; select conserved regions; construct vectors; create recombinant proteins, attenuate vaccine strains (149–153); and create nucleic acid-based vaccines (154, 155), which contain specific gene sequences necessary for the *in vivo* expression of selected antigens. Additionally, the identification of such virulence factors enables researchers to selectively remove regions of the pathogen genome and create safer, attenuated strains for use as live-attenuated vaccines. For example, bubonic plague is caused by *Yersinia pestis* and is one of the deadliest diseases known. A variety of killed, whole-cell vaccines have been available since before 1900, but none are currently licensed (156). A number of live, attenuated vaccines have been produced, but concerns regarding reversion to virulence have precluded their widespread use (156). Current efforts have focused on subunit vaccines, with the subunits (typically virulence factors such as the F1 and V proteins, although other such as NlpD, Caf1 have been used) (157) being identified through genomic approaches. These vaccines have several advantages, including increased safety profiles, rapid induction of protective immunity, and a requirement for fewer vaccine doses (158). Unfortunately, Caf1 deletion does not always prevent lethal infection (159), which suggests that it is not essential for virulence. Similarly, although it is widely assumed that LcrV antibodies are necessary for protection, some primate models indicate that this may not be true for pneumonic plague (160). Further work needs to be done to clarify these issues. Another example is Rift Valley fever virus. The Rift Valley fever non-structural protein NSs was identified as a component that could be removed from the Rift Valley fever veterinary vaccine in order to differentiate infected from vaccinated animals (161). Studies found that the NSs protein was a virulence factor and that removal of the protein increased animal survival from 50 to 95% (162). Virulence proteins can also be used as components of protein-based vaccines. Excellent examples of this are the diphtheria and tetanus toxoid vaccines that contain formaldehyde-detoxified toxins (163), which enable recipients to develop antibodies that recognize and neutralize the native toxins, thus eliminating the major cause of pathogenesis during infection. Sequencing studies have also identified the role of gene sequences in meningococcal antigen expression (164), have identified meningococcal genotypes associated with increase virulence or invasion (165, 166), and have provided insight into immune evasion mechanisms (167).

For a complex pathogen such as plasmodium, in which multiple life-cycle stages occur with very different genes (and proteins) expressed at each stage, it is important to identify the proper sets of immune targets for vaccine development. Genomic technologies have allowed investigators to “mine” the plasmodium genome for antigen discovery. In a recent study, investigators identified the UIS3 gene as essential for parasite development in the liver. UIS3-deficient sporozoites were created and found to infect hepatocytes but were unable to establish a blood-stage infection (168). Vaccination with these modified sporozoites could protect immunized animals from an infectious challenge. In another study, scientists identified genes preferentially expressed by parasites capable of infecting the placenta through the CSA receptor (169). Just like the UIS3 example, these genes may serve as useful targets for a vaccine against malaria in pregnant women. Fortunately, these approaches can also be applied to less complex pathogens. A similar microarray-analysis approach identified Neisseria serogroup B genes that were upregulated during infection and were subsequently demonstrated to encode proteins targeted by protective immune responses (170).

## Barrier #4: New Vaccines and Vaccine Safety

Drivers for the use of genomics in vaccinology include not only the public health need for new vaccines, but also the need to ensure vaccine safety and the need to develop directed approaches to de-risk the costs and time involved in vaccine development. The recognition that human genetic diversity leads to variations in infectious disease expression, severity, and disease outcomes, as well as variations in vaccine response, means that immune responses to vaccines are, at some level, predictable (7). In 2007, we developed and published the immune response network theory, which stated that immune responses to a vaccine are the “cumulative result of *non-random* interactions with host genes, epigenetic phenomena, metagenomics and the microbiome, gene dominance, complementarity, epistasis, co-infections, and other factors occurring within the system as a whole” (7, 92). Critical to our understanding of how vaccines induce protective (or aberrant) immune responses are the ideas that such responses are not random (and hence can be predictable) and occur at the systems level (92). In turn, this led to the development of vaccinomics and systems vaccinology (7, 13, 171–178). This emerging paradigm is an approach that utilizes the tools and insights derived from systems biology; high-dimensional, high-throughput “omics” technologies; and genomics (7, 13, 59, 66, 79, 175, 179–183). Vaccinomics leverages high-resolution data, such as transcriptomics, proteomics, and metabolomics/lipidomics/glycomics, epigenetics, etc., to derive holistic (systems-level) and mechanistic models of both protective and aberrant immune system responses (i.e., “immune signatures”). Such high-dimensional data are utilized in a new, directed four-step vaccine-development paradigm we have described as, “discover, characterize, validate and apply” (13). The idea is to discover new vaccine targets through the use of genomic technologies, characterize these targets in terms of their ability to generate protective immune responses, validate the findings in genetically diverse populations, and apply such findings to new vaccine development and vaccine safety studies.

The increased public scrutiny of vaccine safety has led to several large-scale initiatives designed to enhance our understanding of what drives adverse events after vaccination. One such effort, the BIoVacSafe Project (http://www.biovacsafe.eu/), began in 2012 with an overall goal to improve vaccine safety monitoring and understand what drives adverse reactions to vaccines. The effort had several objectives: (1) to understand early inflammatory responses after vaccination; (2) to develop biomarkers of autoimmunity; and (3) to capture the incidence of autoimmune disease in the population in order to identify those at higher risk of severe adverse events such as anaphylactic shock. A key to this endeavor has been the use of high-dimensional systems vaccinology approaches (184).

Conventional vaccines to prevent infectious diseases typically consist of killed or attenuated pathogens or of proteins from those microorganisms. In contrast, new vaccines being developed, which are poised to make major inroads in medicine, take advantage of genomic technologies to understand which host genes are activated/silenced, which host proteins or metabolites are involved, and what leads to a long duration (durability) of the immune response in vaccinated individuals (O'Connor et al., 119). The second genomic revolution in the vaccine field has to do with the vaccine constructs themselves. Specifically, genomic universal influenza virus vaccines can take the form of DNA or RNA that encode desired hemagglutinins or domains thereof. On administration, the genes enter cells, which then produce the proteins/components of proteins of interest. Compared with manufacturing proteins in cell cultures or whole viruses in embryonated eggs, producing just DNA/RNA is possibly simpler and less expensive. The latter approach is also amenable to making combinations of different epitopes and antigens for complex novel influenza virus vaccines. Checkpoint inhibitors may be used to enhance the immune responses of immunosubdominant epitopes. Finally, genomic vaccines may express antibodies for passive immunization instead of antigens to allow for rapid protection in the case of an emerging pandemic.

ADITEC Project (https://www.aditecproject.eu/) is a European initiative to organize the use of systems biology, adjuvant discovery, immunization routes, novel vaccine vectors and formulations, information about host factors, and results from animal models in order to develop novel immunization technologies and drive vaccine discovery. This consortium has published dozens of papers every year since its inception in 2011 and holds seminars and advanced courses in fields related to vaccinology. This project has resulted in nearly three dozen new immunization technologies, over 20 new animal and *in vitro* models being developed, multiple patents, and at least a dozen clinical trials. In the United States, the Human Vaccines Project is using systems biology, artificial intelligence (AI), and cutting-edge technologies to understand how the immune system functions and responds to vaccines (44). Addressing this fundamental gap in our knowledge will enable us to decode the human immune system, develop predictive markers of vaccine response, and create AI models of the immune system. These and other similar initiatives demonstrate the power of sustained collaborative partnerships between academia, industry, and governmental agencies. Increasingly, sophisticated computational modeling and machine learning approaches will be leveraged to understand immune function (185), identify optimal epitopes (186), as well as design and test new vaccines (187–189).

While the idea of personalized medicine is making progress, very little is known why some humans are more resistant to a pathogen and others are more susceptible. Combined with a better understanding of who responds well to a particular vaccine, this knowledge will be crucial to provide adequate protection and to design novel vaccines/gene sequences for an individual.

Vaccine safety is also being addressed using genetic approaches—termed “adversomics”—using the tools of immunogenomics, systems biology, computational modeling, and bioinformatics in order to better understand both genetic and non-genetic drivers of aberrant vaccine responses at the molecular level (7, 171, 172, 176). This is similar to the use of “omic” technologies in the field of toxicology (190, 191).

Adversomics presupposes that vaccine adverse reactions and events are not random and are predetermined genetically and in other ways. Immune-mediated vaccine adverse events are the primary outcomes of interest for the field of adversomics (184, 192). New biologic understandings, and the necessity of preventing serious adverse vaccine events, are critical to enhancing and—in some population groups—restoring public trust in vaccine safety, and for creating new knowledge applied to developing new vaccines that are both safe and effective. The pathway to accomplishing these goals is to understand the genetic and molecular mechanisms that determine inter-individual variations in vaccine response and reactivity. In turn, mechanistic knowledge of underlying vaccine adverse events could allow the ability to predict serious adverse events, and to design new vaccines that reduce or even eliminate harmful vaccine-related reactions. This endeavor is likely to be complementary to a more individualized approach to vaccine practice.

Examples of the value of genomics in vaccine safety have been published. McKinney et al. identified an association between specific cytokines after smallpox vaccination and the development of fever (193). Stanley et al. identified the influence of specific SNP haplotypes in the IL-1A, IL4, and IL18 gene complex in the development of fever after smallpox vaccination (194). Feenstra and colleagues identified a variety of genes and SNPs [IFI44L, CD46, SCN1A, 2A, TMEM16 (ANO3)] in the etiology of fever and febrile seizures after MMR vaccination (195). We and others have published on the association of myopericarditis after smallpox vaccine (196–200). This has resulted in studies attempting to determine possible genetic associations (176, 201, 202).

## Barrier #5: Non-heritable Factors (e.g., Environment and Geography)

In addition to host genetics, non-heritable or environmental factors (e.g., pathogenic and symbiotic microorganisms, infections, diet, smoking, geographic, and other factors) play a role in shaping biological post-vaccination responses; however, the contribution of environmental factors to vaccine-induced immune responses is less understood (93). It is possible that inter-individual variation in immune responses induced by environmental factors would be significant in shaping adaptive post-vaccination responses. As an example, by using a systems vaccinology approach to assess immune responses stimulated by trivalent inactivated influenza vaccination (TIV), the gene expression of *TLR5* at day 3 after vaccination was found to correlate with influenza vaccination response (HAI titers) 28 days after vaccination (203). While TLR5 mediates the sensing of flagellin on bacteria, it has been shown that it is also necessary to generate B cell responses and Ab production to viral vaccines (e.g., inactivated influenza and inactivated polio vaccines) (203, 204). Vaccination of *TLR5*^−/−^ mice with TIV has caused a substantial reduction in Ab levels and frequencies of short-lived plasma cells confirming the gut microbiota can influence the heterogeneity in vaccine responses. Hence, there is close interaction among the components of the human immune system and the host microbiota, and this interface may influence vaccine-induced immune response and affect vaccine efficacy. Such findings require systems-level omics technologies to dissect the contributions and inter-relationships between multiple factors.

The effect of genetic contribution (heritability) on vaccine-induced immunity has mainly been estimated through monozygotic and dizygotic twin studies, which provided an approach to control for common environmental factors. Most of these studies have found that immune responses to many vaccines are heritable (205–207). For example, the estimated heritability for anti-HBs Ab concentrations after receipt of hepatitis B vaccine ranged between 61 and 91% in different studies (205, 206, 208). With respect to MMR vaccinations, the estimated heritability for Ab responses to measles, mumps and rubella virus vaccines has been found to be 88.5, 38.8, and 45.7%, respectively (209). Using the frequency of the human immune cell repertoire by FACS, a large genetic study of 1,629 individuals (14–102 years old) from Sardinia, Italy, found many cell populations (that are positive for the CD93 marker) with very high heritability (>60%), including Tregs and their subsets (mean 55%) (210). Thus, circulating immune-cell phenotypes may have measurable heritable components. In contrast, a recent systems-level influenza-vaccine twin study by Brodin et al. used 210 healthy twins (8–82 years old) to examine 204 different parameters of the immune system, and immune response outcomes found that non-heritable factors had a greater influence than heritable factors (211). Given that many earlier vaccine investigations in twins have studied infants and young children, the authors proposed that “many if not most of the less heritable traits that we measured in our mostly adult population may be much more heritable if measured in young children” (211). Similar environments may thus falsely suggest heritable traits in vaccinations of twins. It was suggested that variation in the human immune response increases with age and is driven by non-heritable factors, such as frequent environmental contact with various pathogens (e.g., CMV, influenza) and microbes. This hypothesis illustrates one of the challenges in translating findings from genetic studies (e.g., genetic variants that underline heritable immune response traits) to new vaccine development without accounting for continuously changing, non-heritable influences.

## Conclusions and Perspective

Vaccine development in the twenty-first century is enabled by increasingly sophisticated genetic and high-dimensional assays, aided by bioinformatics approaches (212–214). This has allowed unprecedented resolution, at the whole-systems level, of how innate, adaptive, and cellular immune responses are generated, interact, and are maintained after vaccination. These technologies are being further leveraged in understanding adverse (aberrant) vaccine responses and the durability of immunity to vaccines, which represent areas of intense investigation due to their importance to human health. Taken together, genetic technologies and approaches have led to a new era of genetic design of vaccines and have provided solutions to the barriers currently impeding progress in this area (Table 1).

**Table 1:** Genomics-based solutions to vaccine development barriers.

**Barrier**	**Potential solution(s)**
**Understanding how immunity develops** • Poor immunogenicity and/or durability • Lack of correlates of protection • Inefficient activation of innate immunity • Lack of animal models with predictive value	Systems biology studies Identify non-humoral correlates of protection Better understanding of the effector functions associated with spontaneous resolution of infection Vaccines inducing cellular immunity Laboratory assays measuring functional responses correlated with clinical protection
**Host variability** • Inter-individual variability in vaccine response • Non-responder populations • Sex, age, race, ethnic differences in response	Age, sex, or population-based vaccine formulations Diagnostic tests to predict vaccine response
**Pathogen variability** • Pathogen diversity • Antigenic drift and antigenic shift • Hypervariable viruses • Complex biology (e.g., Plasmodium) • Active vs. latent vs. chronic infection • Host pathogen interactions • Immune response evasion mutants	Vaccines eliciting broadly neutralizing Abs Multi-valent vaccines eliciting high affinity Ab to multiple serotypes Universal vaccine based on genetically conserved epitopes Vaccines targeting pre-erythrocytic, blood, and/or mosquito stages Interventions that mitigate pathogen immunomodulation during immune response to vaccination DNA vaccine targeting T cell responses to the partially conserved NS3 and C genes and Ab responses to the E protein
**Vaccine safety** • Adverse events • Autoimmunity • Vaccine hesitancy	Subunit, protein, and peptide-based vaccines incorporating novel adjuvants driving immunogenicity and durable protection Dose-sparing approaches
**Environmental and geographic factors** • Poor nutrition/obesity • Co-infection • Prior immunity • Pollution	DENV-vaccines for naïve and DENV-exposed individuals Vitamin supplementation coadministered with vaccination

*Vaccines are the most effective public health tool for controlling infectious diseases. Despite considerable success, there is room for improvement in many current vaccines and there are a large number of new and re-emerging pathogens for which we do not have effective vaccines. Vaccine development faces a number of challenges, many of which are presented here. Developing vaccines to combat current and future pathogens will require us to overcome those challenges and recent developments in genomic technologies may provide the solutions that we need. Several potential solutions for each barrier are listed in the table while real-world examples are discussed in the text*.

These novel approaches have been driven by public health urgency, demand for vaccine safety, cost considerations, and the inability of past vaccine-development paradigms to lead to viable vaccine candidates against complex and hyper-variable pathogens quickly enough to meet public health needs at an affordable cost. As a result, vaccine development is being accelerated by genetic and bioinformatics approaches (186). In the last decade, new vaccines against influenza have been developed and licensed, as have vaccines against meningococcus group B, hepatitis B, and herpes zoster using genomics-based approaches. Many more vaccines are in development.

Genetic approaches have enabled the identification of relationships/networks between individual genetic variants and specific aspects of vaccine-induced innate, adaptive, or cellular immune responses. The promise of vaccinomics is to identify specific immune response profiles that may serve as signatures or biomarkers that accurately predict vaccine immunogenicity, efficacy, and/or safety. Furthermore, it has the potential to identify genetic variants or antigens that lead to newer and safer vaccine candidates. We believe that the development of very large and detailed genotype:phenotype databases will eventually lead to a new model of personalized vaccine practice (i.e., the delivery of the right vaccine to the right person at the right time) that utilizes genetic and immune signatures to do the following: develop new vaccine candidates; predict the need for a vaccine and the dose needed to induce protective immunity; and to predict whether a significant adverse effect is likely to occur—in other words, personalized vaccinology.

Yet, barriers remain. Issues of high costs for genetic-based assays, including the cost of analysis and the complexity of such data exist, as well as inertia on the part of current vaccine developers conspiring to delay the full use of these rapidly advancing new paradigms. Funders of research must realize not only the promise of such vaccine development approaches but also the costs. For example, the standard allowable budget for the most common NIH research funding mechanism in the USA, the R01, has not changed in the past 30 years despite massive advances in science and the cost of experiments and statistical analysis over this time period.

Infectious diseases have always been—and always will be—a threat to human health. An excellent example of this is the current COVID-19 pandemic. This demonstrates how easily and repeatedly pathogens can emerge and affect humanity on a global scale. We had ample warning that novel coronaviruses can and do jump species and cause widespread and serious disease in humans. Our efforts to create vaccines against SARS and MERS resulted in products that reached clinical trials but no licensed vaccines. Fortunately, what we learned from those outbreaks has been rapidly applied to the SARS-CoV2 and we have seen clinical trials begin within 5 months of the first reported cases. This is a tremendous achievement. We have no choice but to continue to accelerate our ability to protect ourselves against pathogens that harm and kill. We are poised to do so, and the future is bright. Novel tools and paradigms allow highly directed study at levels of genetics and biology unimaginable just a handful of years ago. An example is that of the CRISPR/Cas9 technology that is revolutionizing genome editing of cells and pathogens; this technology has been used to excise virulence genes and create Pseudorabies virus vaccines (215) and to create duck enteritis virus (DEV) recombinants expressing avian influenza (highly pathogenic H5N1) and duck tembusu virus (DTMUV) antigens. The resulting trivalent vaccine elicits protection against all three duck pathogens (216).

Perhaps Albert Camus said it best in his book *The Plague*:

Everybody knows that pestilences have a way of recurring in the world; yet somehow we find it hard to believe in ones that crash down on our heads from a blue sky. There have been as many plagues as wars in history; yet always plagues and wars take people equally by surprise (217).

## Author Contributions

RK, IO, and GP conceived of the topic. RK drafted the manuscript and organized the figures and tables. IO, PP, and GP revised the manuscript. RK, IO, PP, and GP approved the final manuscript. All authors contributed to the article and approved the submitted version.

## Conflict of Interest

GP is the chair of a Safety Evaluation Committee for novel investigational vaccine trials being conducted by Merck Research Laboratories. GP offers consultative advice on vaccine development to Merck & Co. Inc., Avianax, Valneva, Medicago, Sanofi Pasteur, GlaxoSmithKline, Emergent Biosolutions, and Dynavax. GP and IO hold four patents related to vaccinia and measles peptide research. RK has received funding from Merck Research Laboratories to study waning immunity to mumps vaccine. GP, RK, and IO have received grant funding from ICW Ventures for preclinical studies on a peptide-based COVID-19 vaccine. These activities have been reviewed by the Mayo Clinic Conflict of Interest Review Board and are conducted in compliance with Mayo Clinic Conflict of Interest policies. The Icahn School of Medicine has filed patents on the development of universal influenza virus vaccines (PP).

## Glossary

### Vaccinomics

The integration of immunogenetics and immunogenomics with systems biology, immune profiling, and bioinformatics approaches used in the development and study of new vaccines.

### Adversomics

The study of vaccine adverse reactions using immunogenetics and systems biology approaches to better understand the genetic and non-genetic drivers of vaccine adverse reactions at the molecular level.

### Epigenomics

The study of the complete set of epigenetic modifications on the genetic material of a cell (i.e., the epigenome).

### Genome-wide association studies (GWAS)

The observational study of a genome-wide set of genetic variants in different subjects to examine if any one variant is associated with a trait.

### Systems biology

The study of complex systems in order to understand the networks of interactions and effects of those interactions.

### CRISPR–Cas-9 technology

A gene-editing technology that can be used in the development of vaccines.
